# Specialty grand challenge: existing issues and emerging frontiers in renal endocrinology

**DOI:** 10.3389/fendo.2025.1651141

**Published:** 2025-07-17

**Authors:** A. T. M. Emdadul Haque, Mohammed S. Razzaque

**Affiliations:** ^1^ Preclinical Department, Universiti Kuala Lumpur Royal College of Medicine Perak (UniKL RCMP), Ipoh, Perak, Malaysia; ^2^ Department of Medical Education, School of Medicine, University of Texas Rio Grande Valley (UTRGV), Edinburg, TX, United States

**Keywords:** ADH, FGF23, kidney, endocrinology, phosphate

## Introduction

We are currently in an exciting time to advance the field of renal endocrinology. There has never been a more favorable time to study the molecular and cellular mechanisms underlying renal hormone functions in physiology, pharmacology, and pathophysiology. The availability of advanced research tools provides unique opportunities to dissect the complex regulatory roles of renal hormones. The kidneys are vital organs in the human body; each one is composed of approximately one million functional nephrons. Understanding the precise roles and regulation of kidney-derived hormones and hormones that exert endocrine effects on the kidneys will help in preventing or delaying various renal and systemic diseases, including hypertensive complications. The primary functions of the kidneys include filtration of blood, maintenance of fluid, electrolyte and acid–base balances, controlling blood pressure, and facilitating erythropoiesis by generating erythropoietin. In addition to erythropoietin, kidneys also produce renin and help generate functional vitamin D by converting 25-hydroxyvitamin D to 1,25-dihydroxyvitamin D (1, 2). We briefly discuss the extrarenal functions of these kidney-derived factors below, with an emphasis on the clinical and functional aspects that remain to be elucidated.

## Erythropoietin

Under normal physiological conditions, erythropoietin synthesis is tightly regulated; however, when oxygen levels are low, hypoxia-inducible factor (HIF) promotes the expression, synthesis and release of erythropoietin from the kidney (3), which then binds to erythropoietin receptors on erythroid progenitor cells to enhance their survival, proliferation, and differentiation (4). The molecular feedback mechanism that fine-tunes erythropoietin synthesis and subsequent signaling has not yet been clearly defined. Recombinant human erythropoietin is commonly used to treat renal anemia in patients with chronic kidney disease (CKD), cancer, or those undergoing chemotherapy (5, 6). Of therapeutic importance, exogenous erythropoietin and other erythropoiesis-stimulating agents have been linked to an increased risk of adverse cardiovascular events, including hypertension, stroke, and heart failure. Overcorrection of hemoglobin levels may cause hyperviscosity with increased thrombotic risk and endothelial dysfunction (7, 8). Additionally, the distribution of erythropoietin receptors in cells outside the hematopoietic system has been identified, and their physiological significance remains to be determined. For example, the exogenous use of erythropoietin has the potential to exert proliferative effects on tumor cells, as the expression of erythropoietin receptors is detected on tumor cells (usually at low levels) (9, 10). Careful monitoring is necessary when recombinant erythropoietin is used in tumor patients.

## Renin

Renin is another important kidney-derived hormone and is secreted by juxtaglomerular cells in response to hypotension (11). It is an essential component of the renin–angiotensin–aldosterone system, which regulates blood pressure, fluid, and electrolyte balance. The secretion of renin is tightly controlled by baroreceptors, macula densa cells, and sympathetic nerve endings (12). Dysregulation of the renin–angiotensin–aldosterone system, especially excess renin or angiotensin II activity, has been implicated in the development of hypertension, heart failure, and CKD (13). Since the (pro)renin receptor binds both renin and prorenin independent of angiotensin II, the *in vivo* physiological role of the (pro)renin receptor beyond angiotensin generation is unclear. Importantly, renin primarily acts systemically through angiotensinogen cleavage in the circulation; however, the biological significance of tissue-specific renin–angiotensin systems (e.g., the brain, heart, adipose, and immune systems) is not yet clearly understood. It has been observed that tissue-specific renin-angiotensin systems can exert protective effects by activating an anti-inflammatory regulatory pathway, which is mediated through angiotensin II type 2 receptor (AT2R) signaling (14). Additionally, the underlying mechanisms of low-renin-mediated essential hypertension need further mechanistic clarification.

## Calcitriol

Normal kidney structure and function are essential for the production of calcitriol (1,25-dihydroxyvitamin D) via renal 1α-hydroxylase activity. Vitamin D is a pleiotropic hormone that has both skeletal and extraskeletal functions. Vitamin D enables the intestinal absorption of calcium and phosphate to support the mineralization process of the bone and helps maintain the homeostatic balance of serum ionized calcium and phosphate. Vitamin D is also believed to exert immunomodulatory effects on both the innate and adaptive immune systems (15). The replacement of exogenous vitamin D is a common clinical practice in patients with CKD and skeletal disorders. Traditionally, calcitriol has been used to manage secondary hyperparathyroidism in patients with CKD. However, concerns about its association with cardiovascular calcification, along with the availability of selective vitamin D receptor activators (e.g., paricalcitol) and calcimimetic drugs, have introduced alternative treatment options. Additionally, the benefits of calcitriol for bone health, particularly osteoporosis and fracture prevention, are not conclusive, as some randomized trials have shown inconsistent effects on fracture risk and bone mineral density (BMD) (16–18). For updated information on vitamin D testing and dosing, readers are referred to a recent Endocrine Society Clinical Practice Guideline (19).

**Figure 1:**
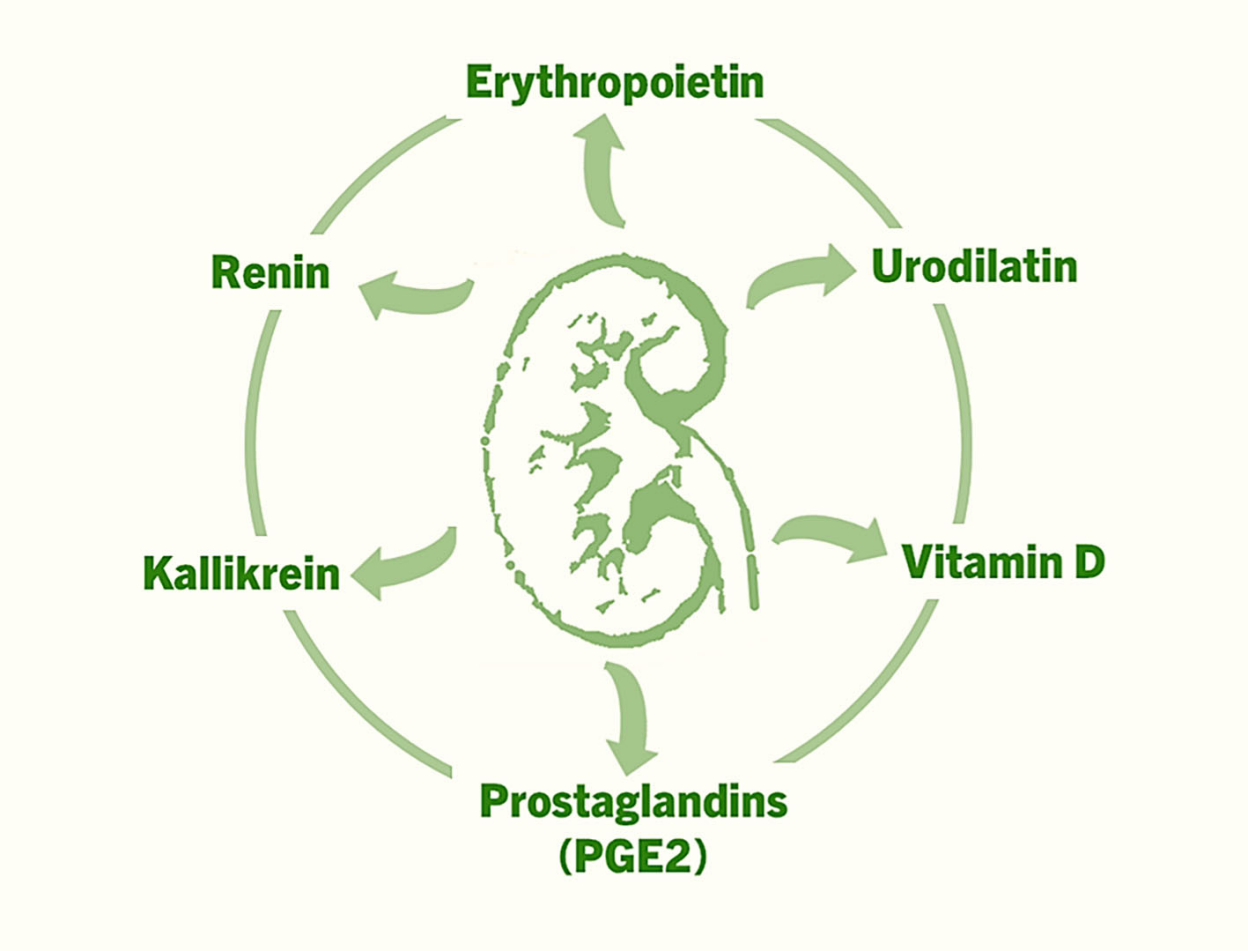
The factors that are produced in the kidneys, and exert autocrine, paracrine and endocrine effects to regulate various functions ranging from controlling blood pressure to facilitating erythropoiesis to musculoskeletal functions.

In addition to the aforementioned factors, prostaglandins (e.g., PGE_2_), kallikrein, and urodilatin also exert autocrine and paracrine effects within the kidney, influencing renal blood flow, sodium excretion (natriuresis), and urine formation (diuresis) (**Figure 1**) (20–22). Normally functioning kidneys are also important for the endocrine effects of hormones produced elsewhere but act on the kidneys. Below, we briefly highlight several key examples, with an emphasis on the unresolved clinical and biological challenges.

## Antidiuretic hormone

ADH (vasopressin) is a hormone synthesized in the hypothalamus that is released from the posterior pituitary gland and acts on the kidneys (23). When plasma osmolality is increased or blood volume is reduced, ADH acts on the collecting ducts of the kidneys to increase water reabsorption, concentrates the urine, and reduces water loss to restore the fluid balance (24). ADH can induce vasoconstriction by interacting with V1 receptors on vascular smooth muscles to maintain blood pressure during hypovolemia (25). The different subtypes of ADH receptors (V1a, V1b, and V2) have distinct tissue distributions and signaling pathways (26, 27). The specific roles of the receptor subtypes in various physiological and pharmacological processes remain poorly understood and require further study to elucidate the delicate functions of the receptor subtypes. Furthermore, ADH and oxytocin are structurally similar peptide hormones, and their receptors have cross-reactivity, indicating that ADH can activate the receptors of oxytocin and vice versa (28). This receptor crosstalk further complicates the interpretation of the behavioral and pharmacological effects of both ADH and oxytocin. Diabetes insipidus (too little or resistance to ADH) and syndrome of inappropriate antidiuretic hormone secretion (SIADH; too much ADH) are the two most commonly encountered clinical conditions that are associated with dysregulation of ADH functions (29). In SIADH, excessive ADH leads to excessive water retention, causing hyponatremia, and in clinical practice, V2 receptor antagonists are used to reduce water reabsorption and promote free water excretion to correct hyponatremia (30). However, the long-term safety of V2 receptor antagonists and their efficacy need further evaluation.

## Aldosterone

Aldosterone is produced by the adrenal gland and exerts its functionality by acting on the distal tubules and collecting ducts of the kidneys. Aldosterone plays a key role in the regulation of blood pressure, electrolyte balance, and fluid homeostasis by promoting sodium retention and potassium excretion in the kidneys (31). On the distal tubules and collecting ducts, aldosterone binds with mineralocorticoid receptors to induce the expression of sodium–potassium ATPase and epithelial sodium channels (ENaCs), increasing sodium reabsorption, potassium secretion, and water retention (32). Collectively, these water and ion redistributions help maintain blood volume and blood pressure. Although the renal effects of aldosterone via mineralocorticoid receptors are well documented, its effects on the cardiovascular and immune systems, as well as neuronal and adipose tissues, need further study, as these receptors are also present in nonrenal tissues, including heart, brain, and fat tissues (33). In addition to aldosterone, cortisol can also activate mineralocorticoid receptors in nonrenal tissues and may exert adverse effects on the heart under stressful conditions or during aging (34). The clinical use of mineralocorticoid receptor antagonists is expanding in conditions such as diabetic nephropathy, heart failure, primary aldosteronism, and liver cirrhosis with ascites. However, further studies are needed to explain why not all patients respond equally to these treatments. Additionally, the regulation of CYP11B2, the gene encoding aldosterone synthase (an enzyme that is necessary for the biosynthesis of aldosterone from precursors such as corticosterone), has yet to be clearly defined in certain clinical conditions, including primary aldosteronism or stress conditions (35, 36). Importantly, angiotensin II and elevated plasma potassium levels are major stimulators of aldosterone release, whereas atrial natriuretic peptide (ANP) inhibits aldosterone secretion (37).

## Atrial natriuretic peptide

ANP is another hormone that is secreted by the atrial myocytes of the heart and affects the kidneys (distal nephrons). In response to hypertension, ANP is released and binds to its receptor-A (NPR-A) to induce vasodilation, an increased glomerular filtration rate (GFR), and sodium excretion to control blood pressure, sodium balance, and fluid volume (38, 39). Furthermore, ANP can suppress the activity of the renin–angiotensin–aldosterone system by reducing the secretion of renin and aldosterone and suppressing sympathetic nervous system activity (40). Recombinant ANP has shown natriuretic, vasodilatory, and neurohormonal-suppressing effects in clinical studies in Japan (41); patients with acute myocardial infarction who received ANP had a smaller infarct size, fewer reperfusion injuries, and better outcomes than controls did. The investigators concluded that ANP could be an adjunctive therapy for patients with acute myocardial infarction who receive percutaneous coronary intervention (41). However, some of the follow-up trials failed to demonstrate such protective benefits (42).

## Parathyroid hormone

PTH plays an essential role in regulating calcium and phosphate homeostasis. PTH is secreted by the parathyroid glands in response to hypocalcemia, hyperphosphatemia and reduced levels of calcitriol (43). PTH acts on distal tubular epithelial cells to increase calcium reabsorption and thereby reduce urinary calcium loss to increase serum calcium levels. PTH inhibits sodium–phosphate cotransporters in proximal tubular epithelial cells to increase urinary phosphate excretion to lower serum phosphate levels. PTH also increases the activity of 1α-hydroxylase in proximal tubular epithelial cells to increase the production of calcitriol, which eventually increases the intestinal absorption of calcium and phosphate (44). In patients with CKD, persistent stimulation of PTH leads to secondary hyperparathyroidism. Although the Kidney Disease Improvement Global Outcomes (KDIGO) working group guidelines recommend maintaining certain levels of PTH in CKD patients, in clinical practice, maintaining such levels is not always easy (45). Additionally, from a biological standpoint, PTH binds to the PTH1 receptor (PTH1R) to exert its bioactivities, and what determines whether PTH1R signals via cAMP pathway or calcium pathway in different tissues is not yet clearly defined (46).

## Calcitonin

Calcitonin is secreted by thyroid parafollicular (C) cells and acts on distal tubules of the kidneys to promote renal calcium excretion by reducing calcium reabsorption in the tubules. Additionally, some reports have shown that kidney cells express both calcitonin and its receptors to perform autocrine and paracrine functions. One of the major issues with calcitonin is its exact *in vivo* role. Importantly, calcitonin deficiency in humans (e.g., post-thyroidectomy) often results in no major disturbances in calcium homeostasis (47–49). Additionally, high serum calcium and gastrin can induce calcitonin secretion, but its feedback control is not yet clear. The presence of calcitonin receptors in tissues not directly involved in calcium regulation, including central nervous system cells and immune cells, requires further studies to dissect the biological role of these receptor complexes. Furthermore, calcitonin secretion decreases with age in both men and postmenopausal women, and whether such a decrease is associated with skeletal age-related phenotypes needs further clarification. Elevated calcitonin levels are detected in the medullary carcinoma of the thyroid gland (50). Clinically, in Paget’s disease of the bone, calcitonin is used to suppress osteoclastic activities and thereby reduce abnormal bone remodeling. The precise role and regulatory effect of calcitonin on renal calcium handling in CKD-related mineral and bone disorders are not yet well defined. Calcitonin, particularly salmon calcitonin, has been used in clinical practice for several conditions related to bone metabolism and calcium regulation. However, in recent days, its clinical use has been limited by the availability of alternative therapies (bisphosphonates or denosumab) with concerns about long-term safety (51).

## Fibroblast growth factor 23

**Figure 2:**
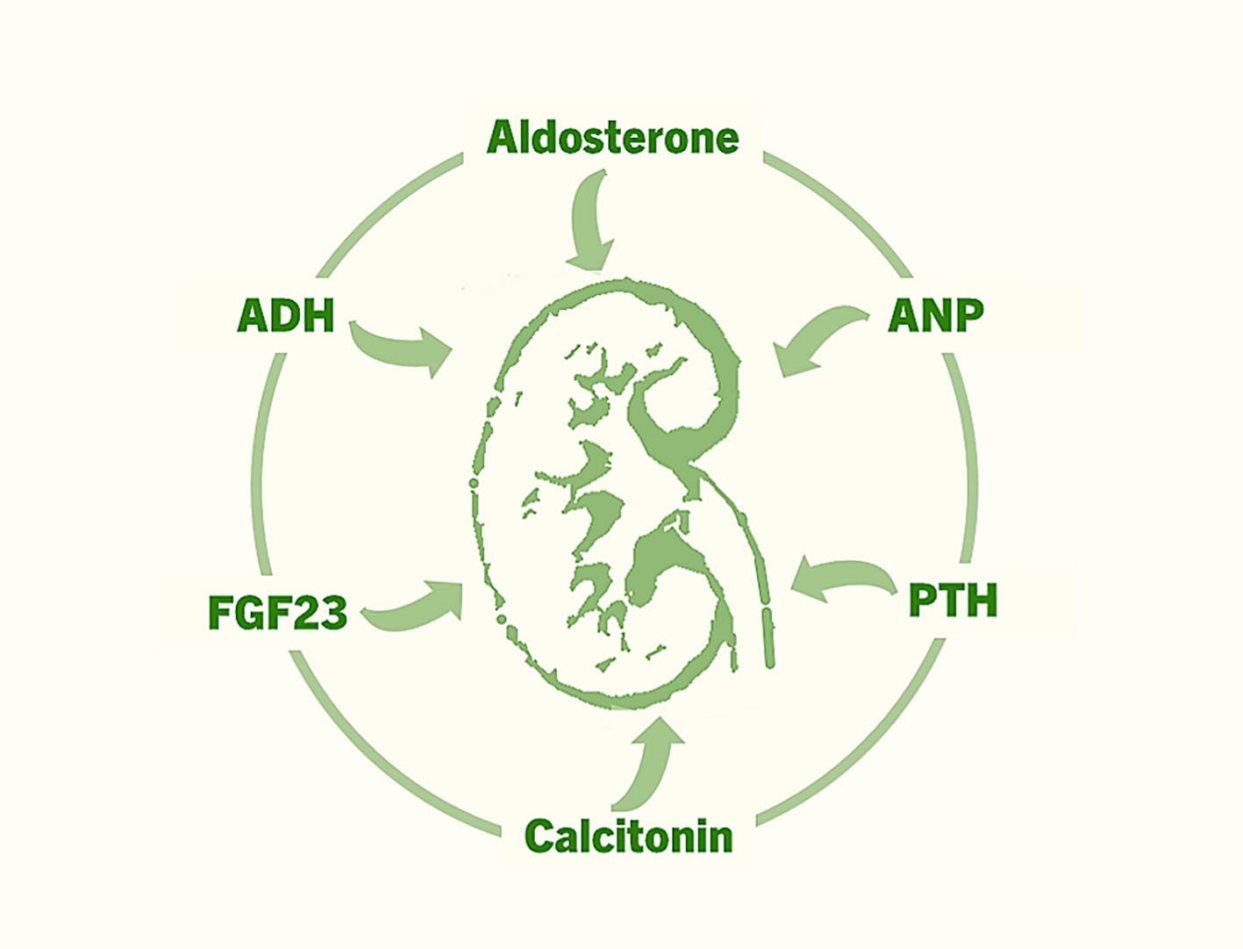
The major hormones that exert various effects on the kidneys to regulate fluid and electrolyte homeostasis, mineral ion balance, acid–base equilibrium, blood pressure, and erythropoiesis.

FGF23 is a relatively recently identified factor that is produced by bone cells and acts on kidney tubules to regulate renal phosphate uptake (**Figure 2**) (52–54). FGF23 reduces renal phosphate reabsorption by suppressing the activities of sodium–phosphate cotransporters (NaPi–IIa and NaPi–IIc) in the proximal tubules, increasing urinary phosphate excretion and resulting in lower levels of serum phosphate. FGF23 also suppresses the production of calcitriol (1,25-dihydroxyvitamin D) by suppressing the renal expression of 1α-hydroxylase (CYP27B1). Phosphate and calcitriol stimulate skeletal FGF23 production, which in turn suppresses renal 1α-hydroxylase expression and phosphate reabsorption, forming a feedback loop that finetunes phosphate and vitamin D homeostasis (55). FGF23 regulates phosphate and vitamin D homeostasis by binding with its cognate FGF receptors (FGFRs) in the kidney, a process that also requires αKlotho and heparan sulfate (HS) as mandatory coreceptors (56). Importantly, αKlotho is synthesized primarily in the kidney. The FGF23-FGFR-αKlotho-HS asymmetric signal transduction unit is responsible for the endocrine regulation of phosphate and vitamin D homeostasis (56). The renal expression of αKlotho is markedly reduced in CKD patients, whereas bone-derived FGF23 expression is upregulated in these patients (57, 58). The exact role of high levels of FGF23 in patients with CKD is an active area of research (59). Additionally, long-term effects of phosphate toxicity because of FGF23 dysregulation need to be resolved (60–63). Clinical and preclinical studies are underway to determine the effects of restoring αKlotho and FGF23 balance in patients with CKD and other diseases with phosphate dysregulation. The identification of FGF23-FGFR-αKlotho interactions provides a structural blueprint for drug discovery to reduce FGF23-linked tissue and organ damage.

## Conclusion

With access to a growing array of molecular and cellular manipulation techniques, including CRISPR-based gene editing, proteomics, genomics, *in vitro* and *in vivo* model systems, we are now better equipped to study the autocrine, paracrine, and endocrine functions of renal hormones, as well as those of hormones that affect the kidney. This presents a unique opportunity to expand our understanding of the mechanisms by which the kidney regulates fluid and electrolyte homeostasis, mineral ion balance, acid–base equilibrium, blood pressure, and erythropoiesis. While the challenges ahead are substantial, we can expect breakthrough research with applied potentials in this rapidly evolving field.
